# Bidirectional causal associations between Sjögren syndrome and gastrointestinal disorders: A two-sample Mendelian randomization study

**DOI:** 10.1097/MD.0000000000050935

**Published:** 2026-09-25

**Authors:** Qiaofeng Wei, Lanlan Li, Qing Du, Hongju Zhang

**Affiliations:** a Department of Rheumatology, Zibo Central Hospital, Zibo, Shandong, China.

**Keywords:** gastric ulcer, irritable bowel syndrome, Mendelian randomization, noninfectious gastroenteritis, Sjögren syndrome

## Abstract

As a systemic autoimmune disorder, Sjögren syndrome (SS) often has symptoms related to the gastrointestinal manifestations. Nevertheless, the causal relationships between SS and gastrointestinal disorders remain incompletely understood. We implemented bidirectional Mendelian randomization (MR) framework to examine causal links between SS and 7 gastrointestinal diseases, including gastric ulcer (GU), duodenal ulcer (DU), irritable bowel syndrome (IBS), Crohn disease (CD), ulcerative colitis (UC), noninfectious gastroenteritis (NGE), and constipation (CP). The inverse-variance weighted approach served as the primary analytical tool while additional techniques used include MR-Egger, weighted median and weighted mode methods. Sensitivity analyses were carried out to determine if there was any evidence of pleiotropy and heterogeneity. Genetic liability for SS was linked with an increased risk of GU (odds ratio [OR] = 1.055, 95% CI = 1.013–1.100) and a reduced risk of IBS (OR = 0.970, 95% CI = 0.948–0.993). Reverse MR analysis indicated that NGE increases the risk of SS (OR = 1.347, 95% CI = 1.118–1.622). Causative relations were not shown between SS and DU, CD, UC, or CP. Sensitivity testing showed stability of the results. This MR analysis presents genetic evidence suggesting that SS increases the risk of GU and decreases the risk of IBS whereas NGE appears to increase the chance of having SS. No causative links with DU, CD, UC, and CP were discovered. These findings support the stratified gastrointestinal monitoring of SS and emphasize the importance of managing gut inflammatory processes within the context of reducing autoimmune risk. However, the protective effect of SS against IBS needs to be investigated.

## 1. Introduction

Sjögren syndrome (SS) is a chronic autoimmune disorder characterized by inflammatory destruction of exocrine glands, resulting in xerophthalmia and xerostomia due to glandular hypofunction. The factors driving SS include genetic predisposition, environmental factors as well as immune dysfunction.^[1]^ Besides the exocrine glands, SS also has an effect on other body organs, including the joints, skin, nervous and digestive systems. Observational studies state that digestive complications occur in 10% to 30% of primary SS patients,^[2]^ suggesting there might be a connection with different gastrointestinal conditions, including gastritis, peptic ulcer, irritable bowel syndrome, Crohn disease (CD), or ulcerative colitis (UC). Observed correlations are interpreted in the context of broader gastrointestinal problems, which could reveal the impact of inflammation on the body^[3]^ and/or hyperactivation of immune processes^[4]^ as well as abnormal activity of specific immune proteins like interleukin (IL)-12B and IL-23R.^[5,6]^ However, these observational findings are often inconsistent and liable to biases like confounding and reverse causality, which limit the ability to support this direct relationship.

Mendelian randomization (MR) is a robust technique that employs genetic variations as instrumental variables (IVs) for eliminating these limitations. This method uses the principle of random distribution of alleles at conception to minimize confounding and help with causal inference.^[7]^ It has been used extensively in establishing causal relationships between SS and different comorbidities of cardiovascular and Parkinson diseases.^[8,9]^ Moreover, various MR studies have concluded the existence of a causal relationship between intestinal microbiome and SS^[10]^ and have pointed out the role of inflammatory cytokines such as FCGR3B and IL-12B in gastrointestinal disorders^[11]^ contributing to the search for possible biomarkers and therapeutic targets. However, a systematic investigation into the causal associations between SS and specific gastrointestinal diseases – such as gastric ulcer (GU), duodenal ulcer (DU), irritable bowel syndrome (IBS), CD, UC, noninfectious gastroenteritis (NGE), and constipation (CP) – remains lacking.

Thus, we conducted bidirectional MR to investigate whether SS has causal links with 7 gastrointestinal conditions: GU, DU, IBS, CD, UC, NGE, and CP. We drew upon publicly available genome-wide association study (GWAS) summary data and applied well-established MR approaches. This allowed us to uncover new evidence on the genetic basis of SS and its gastrointestinal involvement. Taken together, our results provide a genetic viewpoint that could be useful for shaping future treatment strategies and clinical care.

## 2. Materials and methods

### 2.1. Study design and data sources

A two-sample MR analysis in both directions was performed to evaluate the possible causal associations of SS with 7 gastrointestinal disorders (Fig. 1). Associated single-nucleotide polymorphisms (SNPs) were employed as genetic instruments for the risk factors. We complied with MR’s fundamental assumptions^[12]^: first, relevance – SNPs exhibiting robust correlations with target exposures; second, independence – genetic variants unlinked to variables influencing both SS and gastrointestinal outcomes; and third, exclusion restriction – SNPs affecting outcomes exclusively via specified exposure pathways.

**Figure 1.**
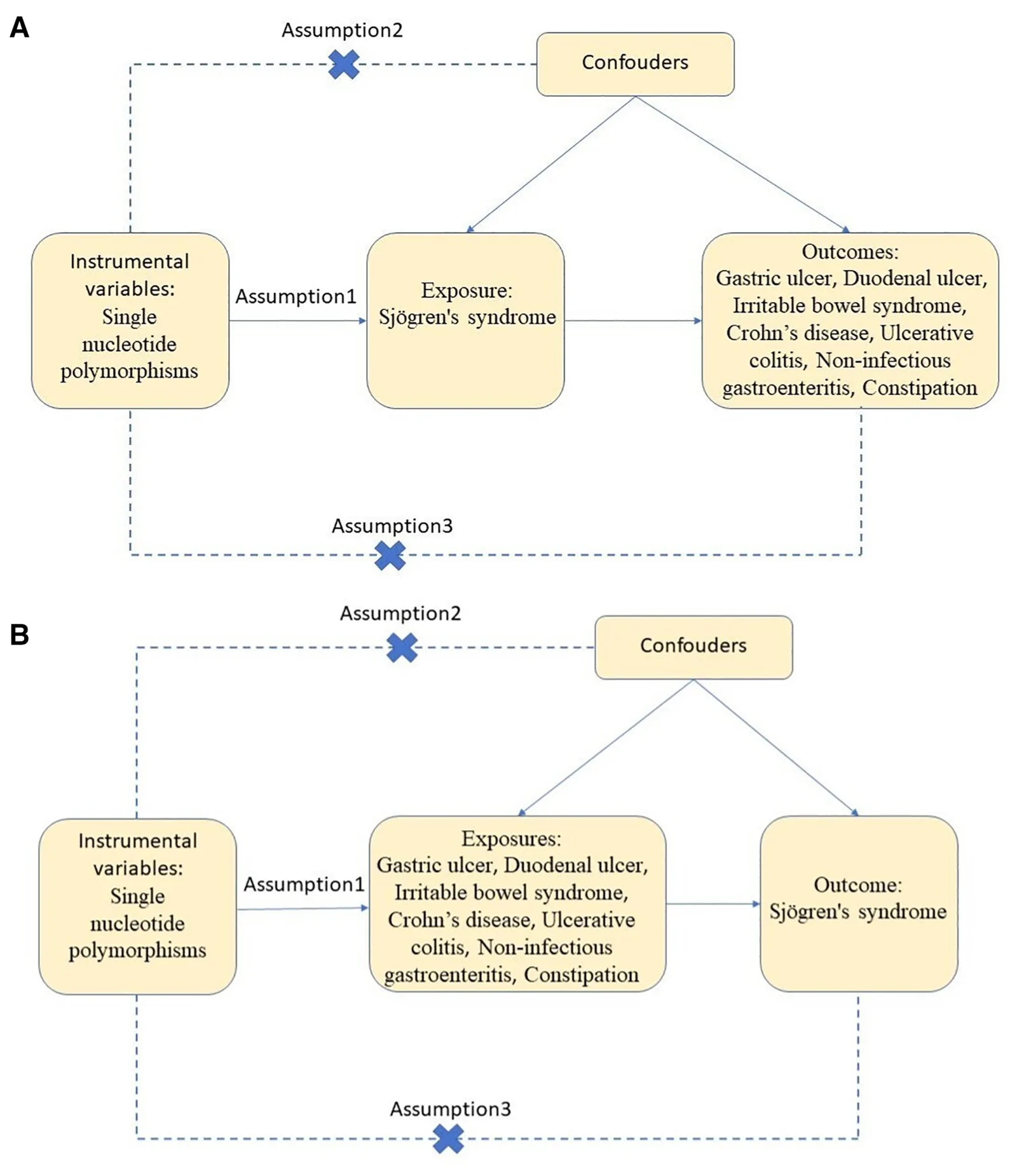
Study design of the bidirectional Mendelian randomization analysis. (A) Mendelian randomization analysis for the effect of Sjögren syndrome on gastrointestinal diseases. (B) Reverse Mendelian randomization analysis for the effect of gastrointestinal diseases on Sjögren syndrome.

GWAS data were acquired from publicly available genetic resources. The characteristics of the cohorts are listed in Table 1. These cohorts only contained participants with European ancestry, enabling the investigators to assure genetic homogeneity in the cohorts. Since these datasets are anonymized and have received ethical approval from their original consortia, the current secondary analysis did not require additional ethical clearance from the institution.

**Table 1:** Characteristics of GWAS cohorts for Sjögren syndrome and 7 gastrointestinal diseases.

Disease	Data source	Cases	Controls	Sample size	Number of SNPs	Authors	Year
Sjögren syndrome	FinnGen Release 12	3309	484,260	487,569	21,306,139		2024
Gastric ulcer	ebi-a-GCST90018851	6293	467,985	474,278	24,178,780	Sakaue et al^[13]^	2021
Duodenal ulcer	GCST90436314	3002	401,525	404,527	28,309,970	Zhou et al^[14]^	2018
Irritable bowel syndrome	ebi-a-GCST90016564	53,400	433,201	486,601	9,739,966	Eijsbouts et al^[15]^	2021
Ulcerative colitis	GCST90468152	–	–	394,626	13,308,322	Loya et al^[16]^	2025
Crohn disease	GCST90468121	–	–	394,626	13,308,322	Loya et al^[16]^	2025
Noninfectious gastroenteritis	GCST90044160	11,373	444,975	456,348	11,831,932	Jiang et al^[17]^	2021
Constipation	ebi-a-GCST90018829	15,902	395,721	411,623	24,176,599	Sakaue et al^[13]^	2021

GWAS = genome-wide association study, SNP = single-nucleotide polymorphism.

### 2.2. IVs selection

To ensure robust instrument strength, we primarily focused on genome-wide significant SNPs that met a strict requirement of *P* < 5 × 10^−8^. However, because of limited SNP availability through preliminary filtering, we applied a more lenient threshold of *P* < 5 × 10^−6,[18]^ so that we could balance statistical power with methodological rigor while maintaining sufficient instrument strength. To address linkage disequilibrium concerns, we clumped the SNPs, introducing a threshold of *r*^2^ < 0.001 and limiting the segments to 10 Mb, retaining only independent variants. Quality control took place afterward, and SNPs with an *F*-statistic < 10 were excluded as weak instruments. Pleiotropy was assessed using the GWAS Catalog and LDlink.^[19]^ Upon the final filtering phase, SNPs that were correlated (*P* < 5 × 10^−6^) with possible confounding factors or alternative pathways were iteratively discarded. During the data harmonization phase, the SNPs that showed suggestive associations (*P* < 5 × 10^−6^) with the outcomes were excluded to avoid reverse causation. All SNPs that were included in the final dataset can be found in Table S1, Supplemental Digital Content 1.

### 2.3. MR analysis

This study adopted multiple MR approaches to strengthen causal inference validity. The major analysis depended on inverse-variance weighted (IVW) techniques since it provides reliable effect estimates assuming balanced pleiotropy.^[20]^ To test the robustness and discover potential pleiotropy, additional analyses including MR-Egger regression, weighted median, and weighted mode methods were performed. All estimates are reported as odds ratios (ORs) and corresponding 95% confidence intervals (CIs). An OR greater than 1 means that there is an increased risk, while an OR below 1 indicates a protective effect.^[21]^

### 2.4. Sensitivity analyses

To confirm the MR estimate reliability, a number of sensitivity tests were carried out. First, we assessed heterogeneity among IVs using Cochran’s *Q* statistic within both the IVW and MR-Egger frameworks; for our tests, we took *P* > .05 to indicate no significant heterogeneity.^[22]^ Second, the MR-Egger intercept test was performed to test for horizontal pleiotropy, whereby *P* > .05 implied that directional pleiotropy was unlikely to be present.^[23]^ Next, the MR-Pleiotropy Residual Sum and Outlier technique was adopted to deal with residual pleiotropy and heterogeneity.^[24]^ This approach systematically identifies and removes outlier SNPs that disproportionately contribute to heterogeneity or pleiotropic effects. Finally, to further assess result stability, a leave-one-out sensitivity analysis was implemented through sequential omission of individual SNPs and recalculation of aggregate effect estimates.^[25]^ This step verified that the inferred causal association remained unaffected by any disproportionately influential genetic variant, thus establishing the robustness of our findings.

### 2.5. Statistical software

All statistical analyses were carried out in R software (version 4.3.1; R Foundation for Statistical Computing) via the two-sample MR package (version 0.6.6; The MR-Base Collaboration).

## 3. Results

### 3.1. Causal effects between SS and gastrointestinal diseases

Initially, we took 24 SNPs identified as linked to SS at the suggestive significance threshold (*P* < 5 × 10^−6^) as our IVs. Through rigorous quality control procedures, SNPs that violated MR assumptions were systematically excluded. These included: 4 SNPs (rs11889341, rs142373084, rs150724213, and rs2004640) associated with systemic lupus erythematosus or rheumatoid arthritis; rs222756, which is linked to CD; and rs35826568, which contains a palindromic sequence. Additionally, rs13211628 was excluded from the NGE and DU analyses due to missing genotype data, and from the UC analysis due to its strong association with the outcome (*P* < 5 × 10^−6^). rs11578282 was not available in the GWAS summary data for CD, UC, or NGE. After making all the exclusions, we ended up with a total of 18 SNPs for GU, IBS, and CP; 17 SNPs for DU and CD; and 16 SNPs for UC and NGE, respectively.

Results from the IVW analysis showed that there exists a causal relationship of SS on an increased risk of GU (OR = 1.055, 95% CI = 1.013–1.100; *P* = .011). Similar estimates were retrieved through complementary approaches: MR-Egger (OR = 1.102, 95% CI = 1.025–1.185; *P* = .022), weighted median (OR = 1.086, 95% CI = 1.029–1.146; *P* = .003), and weighted mode (OR = 1.083, 95% CI = 1.024–1.145; *P* = .015; Figs. 2 and 3). No substantial heterogeneity (Cochran’s *Q* = 14.239, *P* = .357) or directional pleiotropy (MR-Egger intercept = −0.010, *P* = .183) was observed (Table S2, Supplemental Digital Content 2). The symmetry in the funnel plot and testing with leave-one-out sensitivity indicate that these estimates are valid and reliable (Fig. 3).

**Figure 2.**
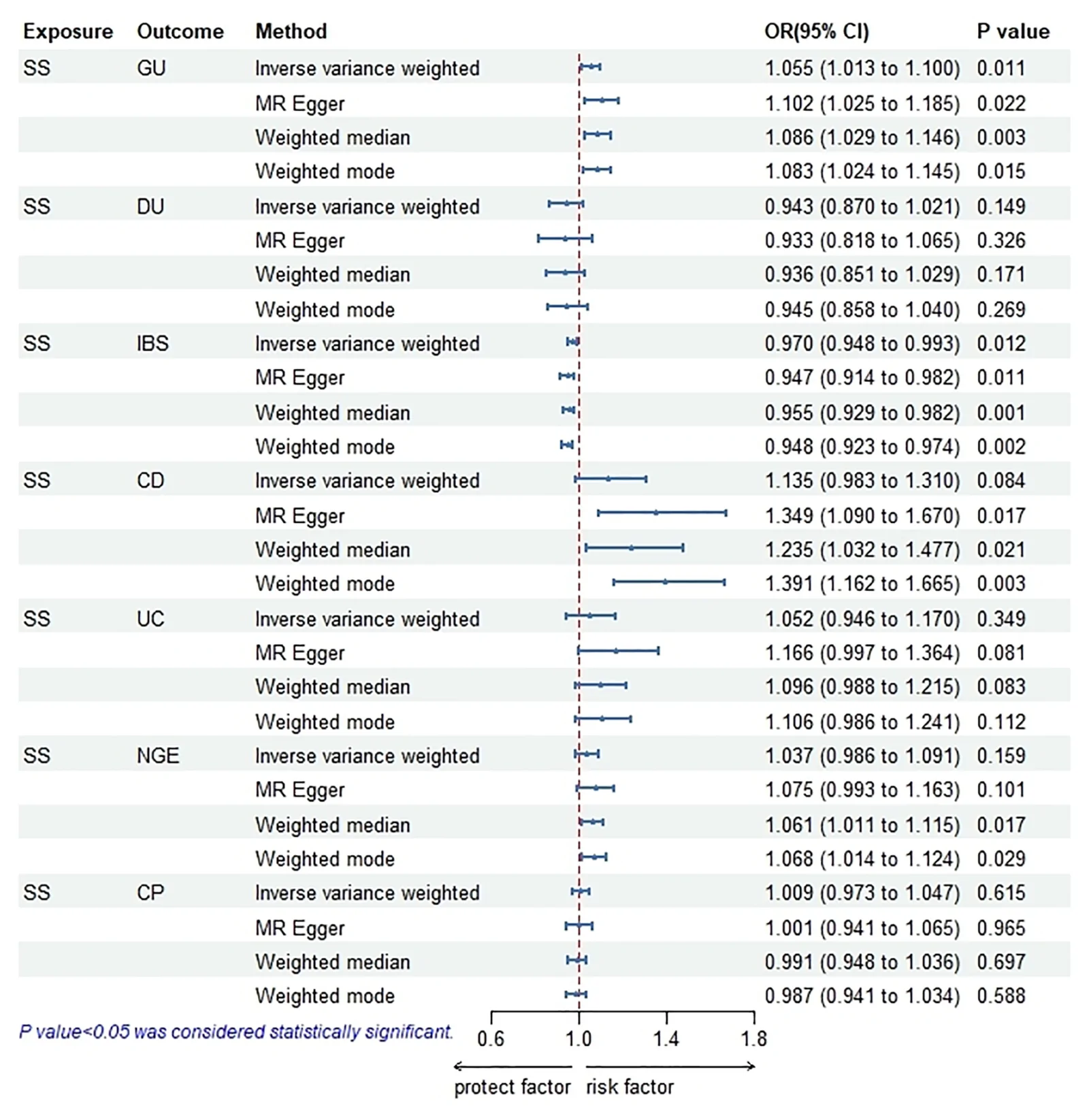
MR analysis for the causal association of SS on gastrointestinal diseases. ORs and 95% CIs derived from inverse-variance weighted, MR-Egger, weighted median, and weighted mode MR analyses are shown. OR > 1 indicates a risk effect, and OR < 1 indicates a protective effect. Statistical significance was set at *P* < .05. CD = Crohn disease, CI = confidence interval, CP = constipation, DU = duodenal ulcer, GU = gastric ulcer, IBS = irritable bowel syndrome, MR = Mendelian randomization, NGE = noninfectious gastroenteritis, OR = odds ratio, SS = Sjögren syndrome, UC = ulcerative colitis.

**Figure 3.**
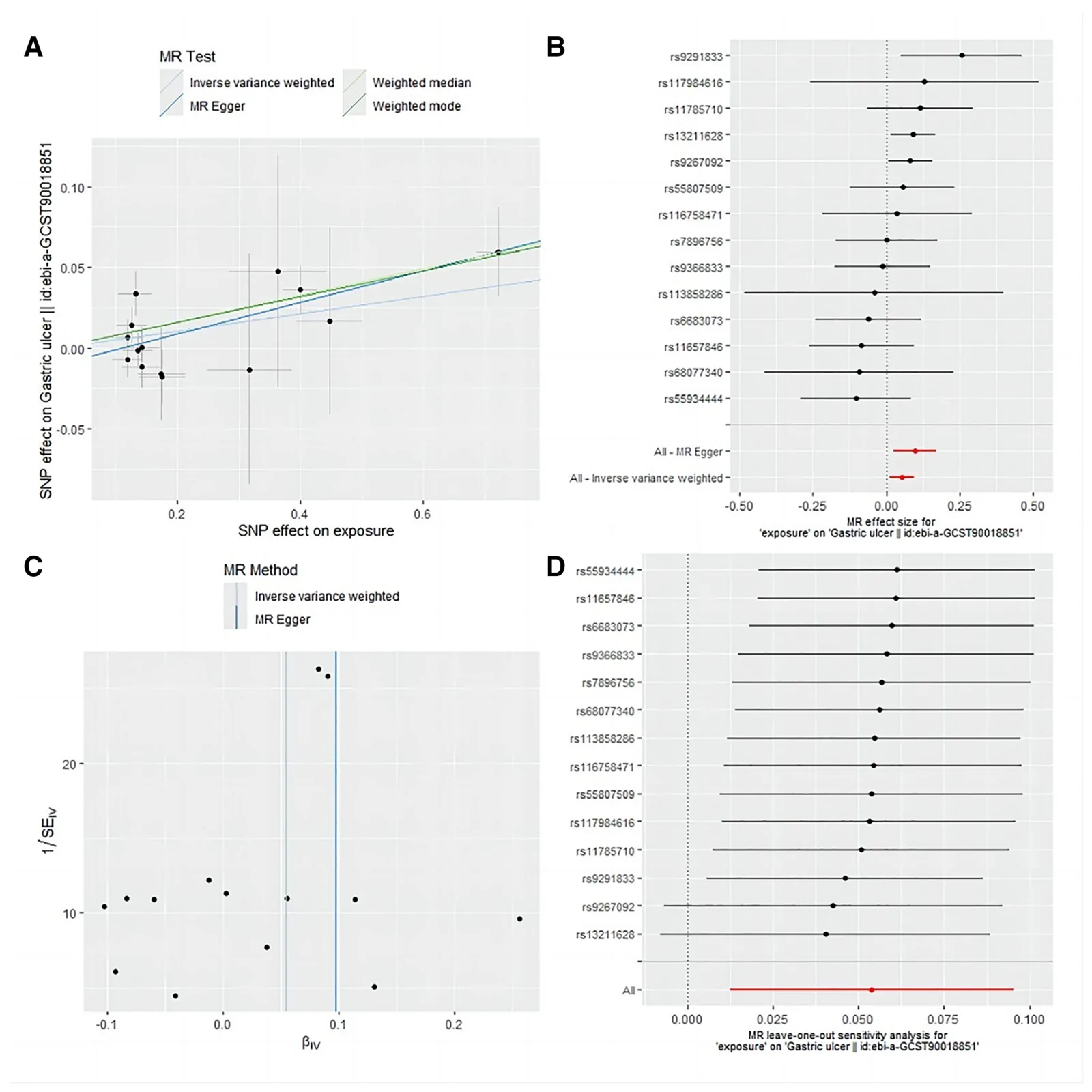
MR and sensitivity analyses of the causal relationship between SS and GU. (A) Scatter plot of SNP effects on exposure versus SNP effects on GU, with regression lines from 4 MR approaches. (B) Forest plot of individual SNP and pooled MR estimates. (C) MR funnel plot showing SNP-specific causal estimates against their precision. (D) Leave-one-out sensitivity analysis to evaluate the robustness of the MR findings. GU = gastric ulcer, MR = Mendelian randomization, SNP = single-nucleotide polymorphism, SS = Sjögren syndrome.

Conversely, genetic predisposition to SS conferred a protective effect against IBS, as evidenced by the IVW analysis (OR = 0.970, 95% CI = 0.948–0.993; *P* = .012). The correlation was further corroborated by MR-Egger (OR = 0.947, 95% CI = 0.914–0.982; *P* = .011), weighted median (OR = 0.955, 95% CI = 0.929–0.982; *P* = .001), and weighted mode (OR = 0.948, 95% CI = 0.923–0.974; *P* = .002; Figs. 2 and 4). Sensitivity analysis assessments did not indicate the presence of heterogeneity (Cochran’s *Q* = 20.165, *P* = .266) or pleiotropy (MR-Egger intercept = 0.007, *P* = .117; Table S2, Supplemental Digital Content 2). Additional validation via funnel plot symmetry and leave-one-out consistency testing further confirmed result reliability (Fig. 4).

**Figure 4.**
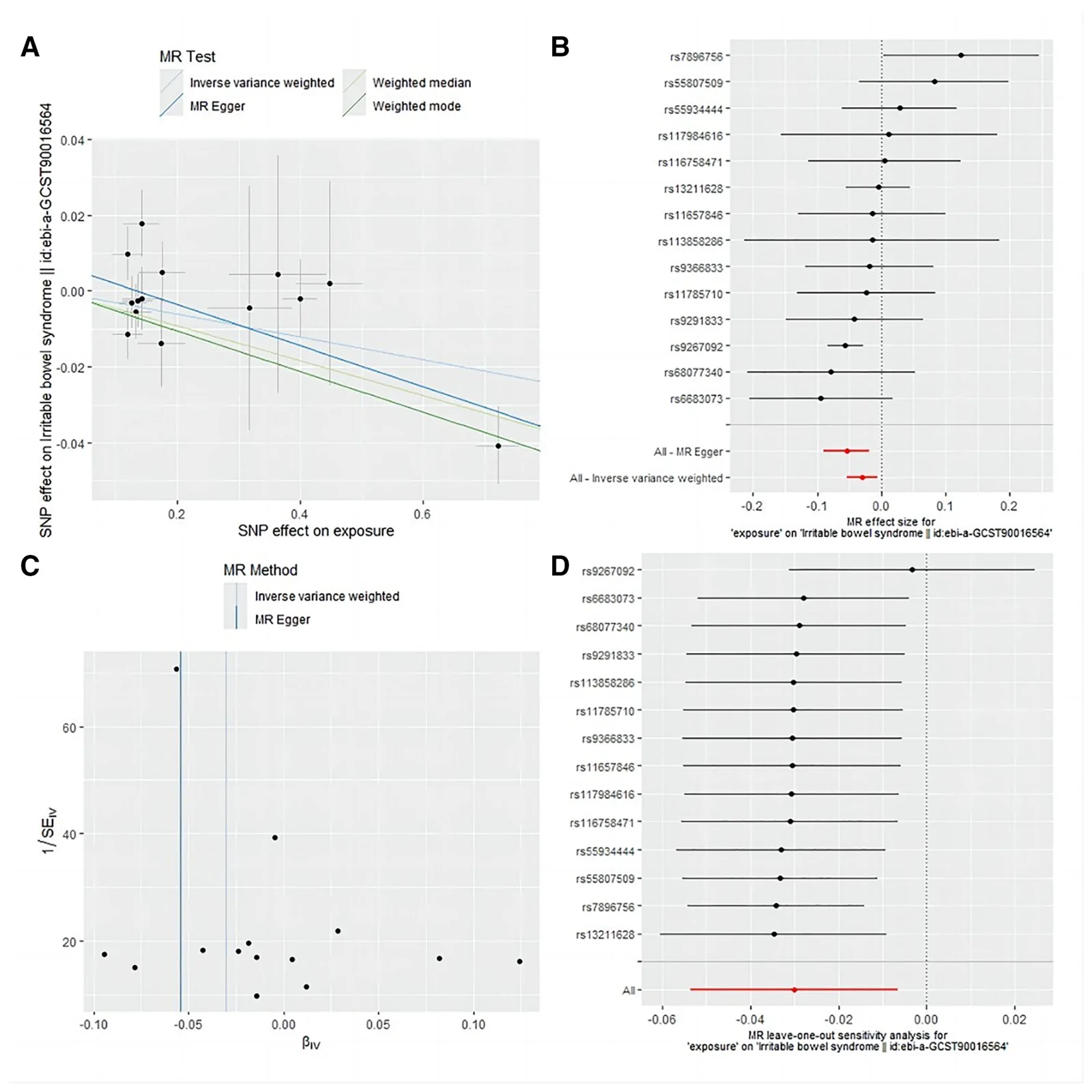
MR and sensitivity analyses of the causal relationship between SS and IBS. (A) Scatter plot of MR analysis showing SNP effect estimates for SS (exposure) versus IBS (outcome). (B) Forest plot of SNP-specific and pooled MR effect estimates of SS on IBS risk. (C) Funnel plot assessing heterogeneity of the SS-IBS MR analysis. (D) Leave-one-out sensitivity analysis verifying the robustness of MR findings by sequentially removing each IV. IBS = irritable bowel syndrome, IV = instrumental variable, MR = Mendelian randomization, SNP = single-nucleotide polymorphism, SS = Sjögren syndrome.

No significant causal effects of SS were observed on DU, CD, UC, NGE, or CP using the IVW method (Fig. 2).

### 3.2. Causal effects between gastrointestinal diseases and SS

This MR investigation evaluated the causal effects of 7 gastrointestinal diseases on SS risk. For GU as the exposure, 30 independent SNPs were initially extracted. During the outcome data merging process, 4 variants (rs111517802, rs505922, rs62647296, and rs78906776) were excluded due to unavailability. Subsequent validation identified 2 SNPs, rs10055792 and rs2976387, as being significantly associated with gastric cancer, leading to their removal from the analysis. At last, 24 robust SNPs were retained for further investigation.

When analyzing DU as the exposure, 16 SNPs were initially extracted. Six SNPs (rs149766138, rs41270957, rs114538624, rs116361371, rs147239450, and rs140022748) were removed due to weak instrument variable status with *F*-statistics below 10. Additionally, rs2976388 for gastric cancer and rs681343 for primary biliary cholangitis were excluded. A final set of 8 SNPs was retained after rigorous quality control.

For IBS as the exposure, 60 SNPs were initially identified. Four SNPs (rs13250534, rs150079703, rs189827057, and rs8092644) were excluded due to absence in the outcome dataset. Further exclusions included rs2736155 (directly associated with the outcome), rs10044618 (linked to Ayurvedic vata prakriti-specific rheumatoid arthritis), rs7106434 (smoking behavior association), and palindromic SNPs rs1036958 and rs541003. This yielded 51 SNPs meeting inclusion criteria.

In the CD analysis, 18 SNPs were initially extracted. Six SNPs (rs114725247, rs145568234, rs146587725, rs146528649, rs7194167, and rs2071142) were excluded as weak instruments. Two additional SNPs – rs2844614 (outcome-related) and rs6878370 (multiple sclerosis-associated) – were also excluded, resulting in 10 SNPs for final analysis.

For UC as the exposure, 38 SNPs were initially identified. Five SNPs (rs112749594, rs11593202, rs11698084, rs11757201, and rs2838516) were excluded due to absence in the outcome dataset. Additional exclusions included rs10995251 (associated with vasculitis), rs145568234 (linked to NGE), rs2836882 (related to primary sclerosing cholangitis), rs3024495 and rs6671847 (associated with systemic lupus erythematosus), rs4655529 (linked to rheumatoid arthritis), and rs7936070 (associated with giant cell arteritis). After rigorous quality control, 26 SNPs were retained for final analysis.

The NGE analysis showed that all 13 SNPs had substantial instrument strengths (*F*-statistic > 10), and there were no instances of pleiotropy or outcome association. This set of SNPs was retained for causal inference without exclusions.

In the analysis of CP as the exposure, of the 17 SNP candidates, only rs139180659 was excluded due to the weak instrument criteria, resulting in a total of 16 SNPs that satisfied the requirements for valid causal estimation.

The IVW analysis revealed a strong causal impact of genetic susceptibility to NGE on SS risk. An increase was established (OR = 1.347, 95% CI = 1.118–1.622, *P* = .0017). Although the supplementary analysis was not statistically significant, the MR-Egger (OR = 1.257, 95% CI = 0.877–1.803, *P* = .239), weighted median (OR = 1.238, 95% CI = 0.956–1.602, *P* = .105), and weighted mode (OR = 1.158, 95% CI = 0.812–1.652, *P* = .434) estimates all aligned directionally with the IVW result, reinforcing the reliability of this association (Figs. 5 and 6). Sensitivity analyses demonstrated the lack of either heterogeneity (Cochran’s *Q* = 12.109, *P* = .437) or horizontal pleiotropy (MR-Egger intercept = 0.009, *P* = .668; Table S3, Supplemental Digital Content 3). The absence of bias was further supported by symmetric funnel plots and consistent leave-one-out estimates (Fig. 6).

**Figure 5.**
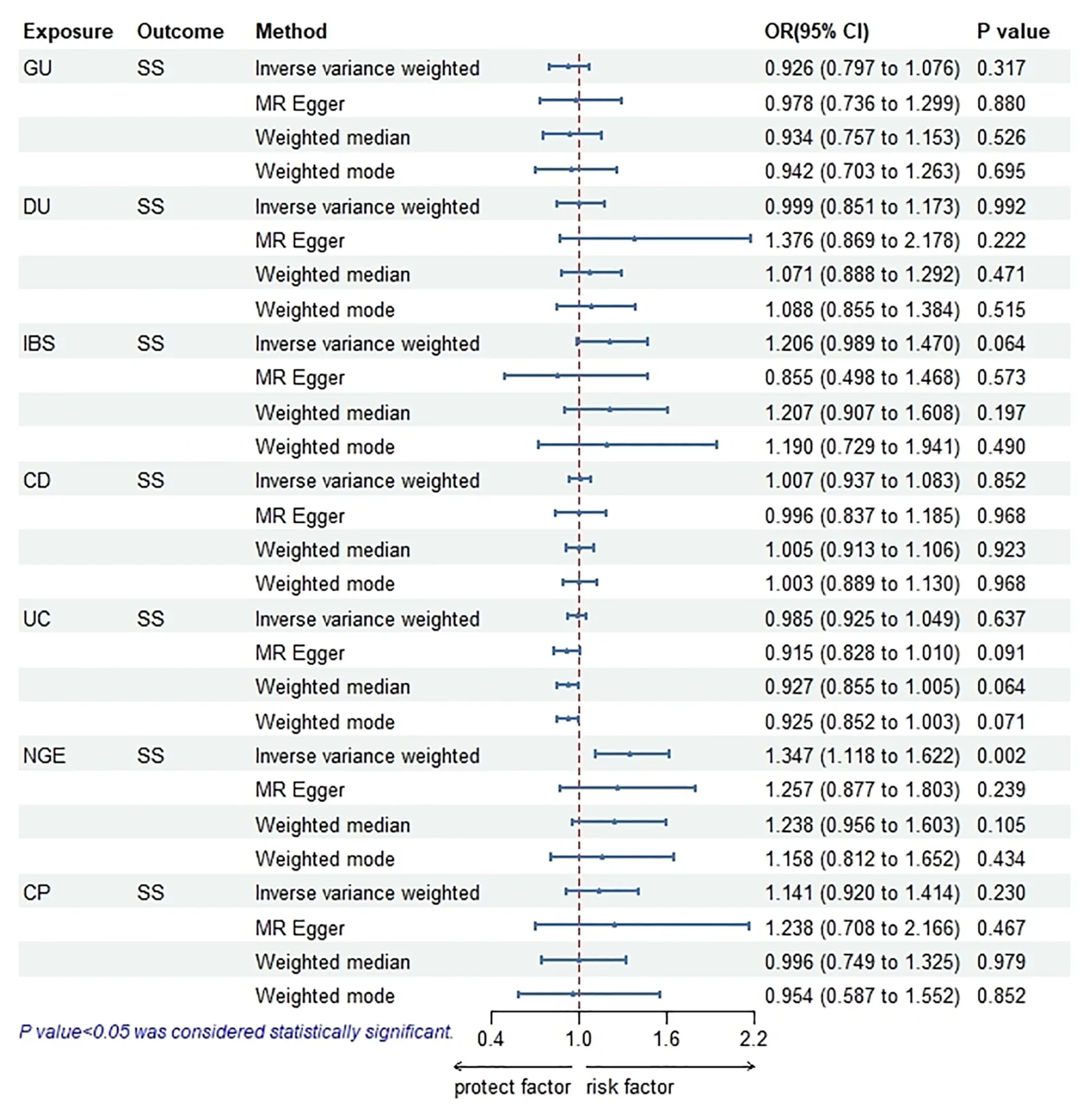
MR analyses evaluating causal associations of gastrointestinal disorders with SS. ORs and 95% CIs derived from inverse-variance weighted, MR-Egger, weighted median, and weighted mode MR analyses are shown. OR > 1 indicates a risk effect, and OR < 1 indicates a protective effect. Statistical significance was set at *P* < .05. CD = Crohn disease, CI = confidence interval, CP = constipation, DU = duodenal ulcer, GU = gastric ulcer, IBS = irritable bowel syndrome, MR = Mendelian randomization, NGE = noninfectious gastroenteritis, OR = odds ratio, SS = Sjögren syndrome, UC = ulcerative colitis.

**Figure 6.**
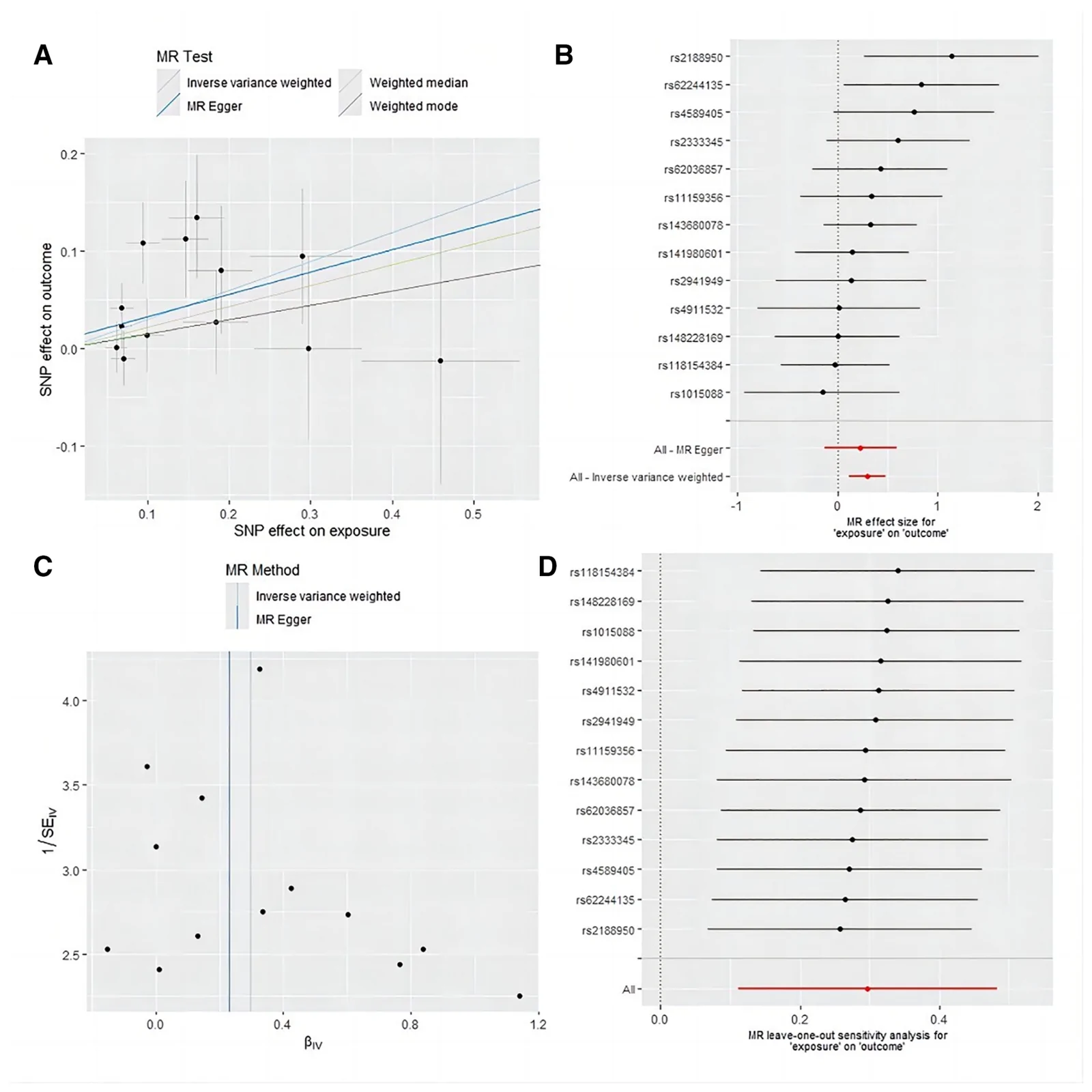
MR and sensitivity analyses for the causal association between NGE and SS. (A) Scatter plot of SNP effect estimates for NGE (exposure) versus SS (outcome), with fitted lines from 4 MR models. (B) Forest plot of SNP-specific and pooled MR effect estimates of NGE on SS risk. (C) Funnel plot evaluating heterogeneity of the NGE-SS MR analysis. (D) Leave-one-out sensitivity analysis verifying the robustness of MR findings by sequentially removing each IV. IV = instrumental variable, MR = Mendelian randomization, NGE = noninfectious gastroenteritis, SNP = single-nucleotide polymorphism, SS = Sjögren syndrome.

To further examine possible reverse causal influence and to test the strength of the causal association between genetic susceptibility to NGE and SS risk, we conducted post hoc sensitivity analyses. Of the 13 instrumental SNPs selected for NGE, 12 passed SNP-level Steiger directionality filtering. One variant (rs2188950) was excluded due to its higher explained genetic variance in SS relative to NGE, indicating potential reverse causality or horizontal pleiotropy. After conducting the Steiger filtering, the causal estimate through IVW remained constant (OR = 1.294, 95% CI = 1.070–1.564, *P* = .0078). The instrument strength *F*-statistic was calculated to be 182, clearly ruling out weak instrument bias. Also, the overall Steiger directionality test provided strong evidence for the causal direction being from NGE to SS (*P* < .001). Post hoc statistical power calculations based on the Burgess framework demonstrated a moderate-to-good statistical power of 74.7% to detect the observed causal effect (OR = 1.294) at the α = 0.05 significance level, supporting the robustness of the primary IVW finding against reverse causation and weak instrument bias.

No significant positive causal associations were identified between the exposures (GU, DU, IBS, CD, UC, and CP) and SS using the IVW method (Fig. 5).

## 4. Discussion

This bidirectional two-sample MR study provides thorough investigations into the causal relationship between SS and 7 gastrointestinal disorders. We examined publicly available GWAS summary statistics, and through an array of genetic IV analyses, we found that genetic susceptibility to SS was associated with an elevated GU risk as well as a lower risk of IBS, although both effects were moderate. In the reverse direction, genetic predisposition to NGE was associated with an elevated risk of SS in the primary IVW analyses. No robust causal associations were detected for DU, CD, UC, or CP in either direction. These findings provide novel genetic evidence clarifying the complex interplay between SS and gastrointestinal disorders and offer useful clinical information on assessing the risk of these health issues.

Our MR analysis suggests that genetic susceptibility to SS may be associated with an elevated GU risk (OR = 1.055), which can be interpreted as a relative risk increase of approximately 5.5%. This association is consistent regardless of the MR model used and passes all sensitivity analyses, which points to a causal dependence. Evidence on the association between SS and GU is scarce. However, data from *Helicobacter pylori* infection help support the theory of the connection, as established in 1 study when patients with SS showed a higher prevalence of *H pylori* (53.83%) compared with controls.^[26]^ In addition, a retrospective study confirmed the association between hypergammaglobulinemia and *H pylori* infection, stating that this condition is an individual predictor of the latter.^[27]^ Besides, more than 50% of patients with SS diagnosed with gastric atrophy had *H pylori* infection,^[28]^ indicating some synergy between infection and SS-associated immune system changes. The mechanistic evidence underlying this association remains largely hypothetical. Chronic lymphocytic infiltration, a hallmark of SS, may result in possible impairment of the mucosal barrier integrity in the stomach and also may contribute to making the gastric mucosa more prone to ulcerogenesis.^[29]^ Another mechanism is linked to autoimmune diseases, exemplified by antimuscarinic type 3 receptor antibodies, which may affect the acid neutralization process.^[30]^ Meanwhile, the presence of pro-inflammatory mediators like tumor necrosis factor-alpha and IL-1β causes systemic inflammation and prolongs the duration of the healing process of ulcers.^[31]^ Decreased efficacy of antioxidant mechanisms may also contribute to oxidative injury of the mucosa via the activation of matrix metalloproteinase-9.^[32]^ Thus, taken together, mechanisms of infection, autoimmunity, inflammation, and oxidative stress can create the conditions for ulcer formation in the stomach. However, it should be understood that these speculations need extra experiments. Clinically, our results indicate that gastrointestinal surveillance should be a part of standard care for those with SS. Regular *H pylori* testing and periodic endoscopy can catch gastric complications earlier and minimize ulcer-related complications.

Our MR findings are in contrast to previous observational studies, which had identified a positive association between SS and susceptibility to IBS.^[33]^ Instead, we demonstrated a protective effect of SS genetic predisposition on IBS (OR = 0.970). This finding was confirmed by additional MR techniques, with no evidence of heterogeneity and pleiotropy. Hence, how could we explain such discordant results? The first obvious explanation is methodological shortcomings. The previous mucosal patch study had only 21 patients, used biomarkers of surrogate type rather than clinical endpoints, and was subject to confounding issues,^[33]^ all of which MR has a high advantage over. However, there may be additional factors affecting the outcome of the study. One of them may be the IBS subtypes because different IBS subtypes may respond differently in terms of inflammation.^[34]^ Another reason may be that the study is based on the given GWAS samples and on the method of population stratification. Another option is to consider that immune dysregulation caused by SS (i.e., disrupted T cell homeostasis, dysregulation of B cells) could have also affected the level of inflammation seen in the intestine.^[35]^ However, all the abovementioned points are just theoretical, since there is no experimental support for any of them. Considering the remarkable nature of the protective association, one should be careful while interpreting it.

Reverse MR analysis indicated an elevated SS risk in relation to NGE genetic predisposition, with no shift in estimates noted after performing the Steiger directionality test. But the claimed link was not fully supported by other MR methods. Therefore, caution is needed when dealing with these findings until they are validated by independent research efforts. If confirmed, these findings would fit into the currently emerging point of view regarding chronic gut inflammation being connected with systemic autoimmunity. Several mechanisms can be proposed to explain the biological rationale behind such a connection. Chronic gastrointestinal inflammation may alter the microbiota, leading to depletion of beneficial metabolites, including butyrate, which is a short-chain fatty acid responsible for regulatory T cell differentiation and inhibiting inflammatory T helper 17 cells processes, consequently helping maintain the balance in immune reactions.^[36,37]^ Additionally, disruption of the gut barrier may allow bacterial products (for instance, lipopolysaccharides), which activate toll-like receptor 4 signaling and enhance type I interferon production, which as it is known, contributes to the pathogenesis of the illness.^[37]^ Another possible factor is that since microbes have antigens that mimic the muscarinic M3 receptor, an autoimmune disease may arise from this interaction.^[30,38]^ Cytokines released from the gut, that is, IL-6 or tumor necrosis factor-alpha may influence B cell leading to the synthesis of anti-SSA/Ro or anti-SSB/La autoantibodies with chemokines recruiting lymphocytes into the gland cells.^[39]^ In this context, all these processes create a vicious cycle that includes dysbiosis, barrier breakdown and inflammation. If proven to be right, the NGE can be treated as the upstream factor of the cause of SS thereby making control of gut inflammation one of the ways of prevention.

Our analysis detected no direct causal link between SS and DU, CD, UC, or CP, nor did we observe significant effects of GU, DU, CD, UC, IBS, or CP on SS risk. These null results contradict previous observational evidence suggesting a link between SS and a wider range of gastrointestinal conditions. One possible explanation is that the previously reported epidemiological links might have been confounded by shared risk factors, including smoking, medication use, and preexisting autoimmune conditions, which cannot be fully accounted for by observational studies. Alternatively, the lack of causal signals may indicate that there is no biological dependence between them, which means that their co-occurrence may be the result of noncausal mechanisms, such as overlapping diagnosis or treatment-related influences.

There are several limitations warrant consideration, despite applying a bidirectional MR design and following strict IV selection and sensitivity analyses. First, we utilized the GWAS summary statistics from a European population; thus, the generalizability of our results outside this group may be limited due to the fact that genetic architecture and genetic studies might vary across populations. Second, it was impossible to stratify the MR analyses in terms of distinct SS subtypes from a clinical or mechanistic point of view, as all genetic data required for such analysis is still unavailable. Third, even though both SS and gastrointestinal disorders show different gender-related differences, we still have not been able to execute a gender-stratified analysis due to restricting data limitations. Fourth, despite the fact that we have run several sensitivity checks, horizontal pleiotropy cannot be excluded completely, since none of the MR methods can ever guarantee this. Fifth, it is possible that cases of SS in the discovery GWAS were not large enough for the study to gain the necessary statistical power to identify small-to-medium-sized effects that were expected in reverse analyses; thus, null findings should be interpreted with caution and do not definitively preclude the existence of modest causal relationships. Finally, the MR approach is based on some assumptions that may not work perfectly; for example, the MR approach does not cover how SS changes over time and across environmental exposure. Despite these limitations, the overall consistency of our results with different MR methods and sensitivity tests enhances the credibility of the detected causal associations and serves as a basis for future mechanistic and clinical studies.

## 5. Conclusion

The bidirectional MR study reveals genetic evidence indicating possible causal associations between SS and 7 gastrointestinal disorders. It is revealed that genetic liability to SS was associated with a moderately elevated GU risk, which indicates that testing for *H pylori* and pathological conditions of stomach can be beneficial for SS patients. On the contrary, it has been established that SS helps prevent IBS contradicting previous observational studies demonstrating the necessity for further research in this field. In the reverse direction, genetic predisposition to NGE was shown to correlate with higher risk of SS. However, due to the fact that this relationship was not consistently documented by other MR methods, the results should be treated with caution and require verification in independent groups. Causal relationships between SS and DU, CD, UC, and CP were not revealed. From a clinical perspective, these findings may support a stratified method in gastrointestinal monitoring of SS patients, while the gut-immune axis warrants further exploration as a potential target for intervention. Future studies, which should incorporate multi-omics data and validation in larger and more diverse populations, will certainly be needed to clarify the biological mechanisms underlying the above-described relations.

## Acknowledgments

The authors want to acknowledge the individuals and organizations that contributed to the Finnish database and the IEU OpenGWAS and GWAS Catalog database.

## Author contributions

**Data curation:** Lanlan Li, Qing Du.

**Formal analysis:** Qiaofeng Wei, Qing Du, Hongju Zhang.

**Investigation:** Lanlan Li, Qing Du.

**Methodology:** Qiaofeng Wei.

**Project administration:** Qiaofeng Wei.

**Resources:** Qiaofeng Wei, Hongju Zhang.

**Software:** Hongju Zhang.

**Visualization:** Hongju Zhang.

**Writing – original draft:** Qiaofeng Wei, Lanlan Li, Qing Du.

**Writing – review & editing:** Hongju Zhang.







## References

[1] Xu J Si S Han Y Zeng L Zhao J. Genetic insight into dissecting the immunophenotypes and inflammatory profiles in the pathogenesis of Sjogren syndrome. J Transl Med. 2025;23:56. 10.1186/s12967-024-05993-z PMC11726950 39806364

[2] Melchor S Sánchez-Piedra C Fernández Castro M. Digestive involvement in primary Sjögren’s syndrome: analysis from the Sjögrenser registry. Clin Exp Rheumatol. 2020;38:110–5. 33025900

[3] Yao YF Wang MY Dou XY. Gastrointestinal microbiome and primary Sjögren’s syndrome: a review of the literature and conclusions. Int J Ophthalmol. 2022;15:1864–72. 10.18240/ijo.2022.11.19 PMC9631199 36404958

[4] Wu G Wu F Wang L. Application of imaging mass cytometry for spatially profiling the microenvironment of salivary glands in primary Sjögren’s syndrome. Cell Death Dis. 2025;16:392. 10.1038/s41419-025-07717-7 PMC12084299 40379615

[5] Ye K Yao X Lu D Tan C Wang X. Common pathological mechanisms and therapeutic strategies in primary Sjogren’s syndrome and primary biliary cholangitis: from tissue immune microenvironment to targeted therapy. Front Immunol. 2025;16:1620073. 10.3389/fimmu.2025.1620073 PMC12515855 41089692

[6] Lee HW Chung SH Moon CM. The correlation of serum IL-12B expression with disease activity in patients with inflammatory bowel disease. Medicine (Baltim). 2016;95:e3772. 10.1097/MD.0000000000003772 PMC4907655 27281077

[7] Ference BA Holmes MV Smith GD. Using Mendelian randomization to improve the design of randomized trials. Cold Spring Harb Perspect Med. 2021;11:a040980. 10.1101/cshperspect.a040980 PMC8247560 33431510

[8] Wang J Li Y Zhang J. Causal relationships between Sjögren’s syndrome and Parkinson’s disease: a Mendelian randomization study. Int J Rheum Dis. 2024;27:e15128. 10.1111/1756-185X.15128 38509724

[9] Liu T Wang L Zheng H Lyu Z Wang B. Effects of Sjogren’s syndrome on essential hypertension: a two-sample Mendelian randomization study. Minerva Cardiol Angiol. 2024;72:588–94. 10.23736/S2724-5683.24.06522-0 38949761

[10] Wang X Liu M Xia W. Causal relationship between Sjögren’s syndrome and gut microbiota: a two-sample Mendelian randomization study. Biomedicines. 2024;12:2378. 10.3390/biomedicines12102378 PMC11505323 39457690

[11] Zhan D Yang Z Li P Pan J. Therapeutic targets for gastrointestinal diseases: proteome-wide Mendelian randomization and colocalization analyses. Postgrad Med J. 2025;101:283–90. 10.1093/postmj/qgae140 39400547

[12] Emdin CA Khera AV Kathiresan S. Mendelian randomization. JAMA. 2017;318:1925–6. 10.1001/jama.2017.17219 29164242

[13] Sakaue S Kanai M Tanigawa Y; FinnGen. A cross-population atlas of genetic associations for 220 human phenotypes. Nat Genet. 2021;53:1415–24. 10.1038/s41588-021-00931-x PMC12208603 34594039

[14] Zhou W Nielsen JB Fritsche LG. Efficiently controlling for case-control imbalance and sample relatedness in large-scale genetic association studies. Nat Genet. 2018;50:1335–41. 10.1038/s41588-018-0184-y PMC6119127 30104761

[15] Eijsbouts C Zheng T Kennedy NA; 23andMe Research Team. Genome-wide analysis of 53,400 people with irritable bowel syndrome highlights shared genetic pathways with mood and anxiety disorders. Nat Genet. 2021;53:1543–52. 10.1038/s41588-021-00950-8 PMC8571093 34741163

[16] Loya H Kalantzis G Cooper F Palamara PF. A scalable variational inference approach for increased mixed-model association power. Nat Genet. 2025;57:461–8. 10.1038/s41588-024-02044-7 PMC11821521 39789286

[17] Jiang L Zheng Z Fang H Yang J. A generalized linear mixed model association tool for biobank-scale data. Nat Genet. 2021;53:1616–21. 10.1038/s41588-021-00954-4 34737426

[18] Zou M Zhang W Shen L Xu Y Zhu Y. Causal association between inflammatory bowel disease and herpes virus infections: a two-sample bidirectional Mendelian randomization study. Front Immunol. 2023;14:1203707. 10.3389/fimmu.2023.1203707 PMC10351388 37465669

[19] Meng Z Ao B Wang W. Relationships between screen time and childhood attention deficit hyperactivity disorder: a Mendelian randomization study. Front Psychiatry. 2024;15:1441191. 10.3389/fpsyt.2024.1441191 PMC11457377 39376970

[20] Lin Z Deng Y Pan W. Combining the strengths of inverse-variance weighting and Egger regression in Mendelian randomization using a mixture of regressions model. PLoS Genet. 2021;17:e1009922. 10.1371/journal.pgen.1009922 PMC8639093 34793444

[21] Xu W Zhang F Shi Y Chen Y Shi B Yu G. Causal association of epigenetic aging and COVID-19 severity and susceptibility: a bidirectional Mendelian randomization study. Front Med (Lausanne). 2022;9:989950. 10.3389/fmed.2022.989950 PMC9538153 36213637

[22] Wu E Cheng M Yang S. Causal relationships of infection with *Helicobacter pylori* and herpesvirus on periodontitis: a Mendelian randomization study. Heliyon. 2024;10:e35904. 10.1016/j.heliyon.2024.e35904 PMC11365429 39220896

[23] Burgess S Thompson SG. Interpreting findings from Mendelian randomization using the MR-Egger method. Eur J Epidemiol. 2017;32:377–89. 10.1007/s10654-017-0255-x PMC5506233 28527048

[24] Verbanck M Chen CY Neale B Do R. Detection of widespread horizontal pleiotropy in causal relationships inferred from Mendelian randomization between complex traits and diseases. Nat Genet. 2018;50:693–8. 10.1038/s41588-018-0099-7 PMC6083837 29686387

[25] Nolte IM. Metasubtract: an R-package to analytically produce leave-one-out meta-analysis GWAS summary statistics. Bioinformatics. 2020;36:4521–2. 10.1093/bioinformatics/btaa570 PMC7750933 32696040

[26] Chen Q Zhou X Tan W Zhang M. Association between *Helicobacter pylori* infection and Sjögren syndrome: a meta-analysis. Medicine (Baltimore). 2018;97:e13528. 10.1097/MD.0000000000013528 PMC6310521 30544455

[27] He Y Hu L Qiu W Zhu L Zhu X Hong M. Clinical characteristics and risk factors of *Helicobacter pylori* infection-associated Sjogren’s syndrome. Immun Inflamm Dis. 2023;11:e994. 10.1002/iid3.994 PMC10614117 37904694

[28] Nishiyama S Ohashi K Aita T Yoshinaga Y. A study of clinical features of atrophic gastritis in patients with Sjögren’s disease. Mod Rheumatol. 2025;35:872–7. 10.1093/mr/roaf031 40119498

[29] Popov Y Salomon-Escoto K. Gastrointestinal and hepatic disease in Sjogren syndrome. Rheum Dis Clin North Am. 2018;44:143–51. 10.1016/j.rdc.2017.09.010 29149922

[30] Namkoong E Lee SW Kim N Choi Y Park K. Effect of anti-muscarinic autoantibodies on leukocyte function in Sjögren’s syndrome. Mol Immunol. 2017;90:136–42. 10.1016/j.molimm.2017.07.007 28750255

[31] Tourani M Habibzadeh M Karkhah A Shokri-Shirvani J Barari L Nouri HR. Association of TNF-α but not IL-1β levels with the presence of *Helicobacter pylori* infection increased the risk of peptic ulcer development. Cytokine. 2018;110:232–6. 10.1016/j.cyto.2018.01.003 29456060

[32] Choudhary P Biswas S Kandoth N. Graphene quantum dots alleviate ROS-mediated gastric damage. iScience. 2022;25:104062. 10.1016/j.isci.2022.104062 PMC8961226 35359805

[33] Lidén M Kristjánsson G Valtysdottir S Venge P Hällgren R. Cow’s milk protein sensitivity assessed by the mucosal patch technique is related to irritable bowel syndrome in patients with primary Sjögren’s syndrome. Clin Exp Allergy. 2008;38:929–35. 10.1111/j.1365-2222.2008.02983.x PMC2440347 18498540

[34] Camargo Tavares L Lopera-Maya EA Bonfiglio F. Rome III criteria capture higher irritable bowel syndrome SNP-heritability and highlight a novel genetic link with cardiovascular traits. Cell Mol Gastroenterol Hepatol. 2024;18:101345. 10.1016/j.jcmgh.2024.04.002 PMC11176963 38643935

[35] Cano-Ortiz A Laborda-Illanes A Plaza-Andrades I. Connection between the gut microbiome, systemic inflammation, gut permeability and FOXP3 expression in patients with primary Sjögren’s syndrome. Int J Mol Sci. 2020;21:8733. 10.3390/ijms21228733 PMC7699261 33228011

[36] Kim DS Woo JS Min H-K. Short-chain fatty acid butyrate induces IL-10-producing B cells by regulating circadian-clock-related genes to ameliorate Sjögren’s syndrome. J Autoimmun. 2021;119:102611. 10.1016/j.jaut.2021.102611 33631650

[37] Cao Y Lu H Xu W Zhong M. Gut microbiota and Sjögren’s syndrome: a two-sample Mendelian randomization study. Front Immunol. 2023;14:1187906. 10.3389/fimmu.2023.1187906 PMC10299808 37383227

[38] Baldini C Chatzis LG Fulvio G La Rocca G Pontarini E Bombardieri M. Pathogenesis of Sjögren’s disease: one year in review 2024. Clin Exp Rheumatol. 2024;42:2336–43. 10.55563/clinexprheumatol/i8iszc 39656593

[39] Qi W Tian J Wang G. Advances in cellular and molecular pathways of salivary gland damage in Sjögren’s syndrome. Front Immunol. 2024;15:1405126. 10.3389/fimmu.2024.1405126 PMC11266040 39050857

