# Supplementary material for: Bidirectional causal associations between Sjögren syndrome and gastrointestinal disorders: A two-sample Mendelian randomization study
Source: Medicine (Baltimore). 2026 Sep 25;105(39):e50935. doi: 10.1097/MD.0000000000050935 (PMC13619203; doi:10.1097/MD.0000000000050935)
Supplement: Supplementary file 1 [file medi-105-e50935-s001.docx]

**Supplementary Table 1** SNPs employed as instrumental variables for two-sample Mendelian randomization between Sjögren's syndrome and gastrointestinal diseases.

SNPs for SS

|  | SNP | Effect allele | Other allele | β | SE | EAF | P value | F-statistic |
| --- | --- | --- | --- | --- | --- | --- | --- | --- |
| 1 | rs113858286 | G | C | 0.318091 | 0.067839 | 0.02875 | 2.75E-06 | 14.73987 |
| 2 | rs11657846 | T | G | -0.14168 | 0.030811 | 0.219128 | 4.26E-06 | 32.45188 |
| 3 | rs116758471 | G | A | 0.447492 | 0.054637 | 0.040568 | 2.60E-16 | 18.30132 |
| 4 | rs11785710 | A | G | 0.125397 | 0.024946 | 0.422526 | 4.99E-07 | 40.08011 |
| 5 | rs117984616 | A | G | 0.363893 | 0.079033 | 0.019961 | 4.14E-06 | 12.65225 |
| 6 | rs35826568 | T | A | -0.1221 | 0.024877 | 0.4667 | 9.19E-07 | 40.19093 |
| 7 | rs55807509 | T | C | -0.11944 | 0.024822 | 0.568762 | 1.49E-06 | 40.27997 |
| 8 | rs55934444 | T | G | -0.17534 | 0.037185 | 0.140258 | 2.41E-06 | 26.88939 |
| 9 | rs6683073 | T | C | 0.119719 | 0.024667 | 0.494485 | 1.21E-06 | 40.53304 |
| 10 | rs68077340 | T | C | 0.174458 | 0.038148 | 0.111744 | 4.80E-06 | 26.21102 |
| 11 | rs7896756 | A | G | 0.142592 | 0.029302 | 0.213098 | 1.14E-06 | 34.12276 |
| 12 | rs9267092 | T | G | 0.72199 | 0.034664 | 0.090842 | 2.39E-96 | 28.84523 |
| 13 | rs9291833 | C | A | -0.13228 | 0.027352 | 0.305403 | 1.32E-06 | 36.55499 |
| 14 | rs9366833 | G | A | -0.13526 | 0.025078 | 0.430371 | 6.90E-08 | 39.86965 |

SNPs for GU

|  | SNP | Effect allele | Other allele | β | SE | EAF | P value | F-statistic |
| --- | --- | --- | --- | --- | --- | --- | --- | --- |
| 1 | rs10076442 | C | T | 0.0705 | 0.0154 | 0.238666 | 4.44E-06 | 64.93479 |
| 2 | rs10823486 | T | G | -0.0628 | 0.0131 | 0.135408 | 1.70E-06 | 76.33556 |
| 3 | rs111467800 | T | C | 0.0568 | 0.0123 | 0.246741 | 3.66E-06 | 81.30047 |
| 4 | rs11175666 | T | C | 0.138 | 0.0287 | 0.113528 | 1.52E-06 | 34.84306 |
| 5 | rs112608939 | T | A | 0.2484 | 0.051 | 0.034921 | 1.11E-06 | 19.60776 |
| 6 | rs116084058 | A | G | 0.1923 | 0.0391 | 0.057962 | 8.98E-07 | 25.57534 |
| 7 | rs11665674 | G | A | 0.0773 | 0.0148 | 0.132989 | 1.94E-07 | 67.56728 |
| 8 | rs12327385 | G | T | 0.1051 | 0.0219 | 0.214379 | 1.66E-06 | 45.66191 |
| 9 | rs12995606 | G | A | -0.0539 | 0.0114 | 0.285724 | 2.30E-06 | 87.71893 |
| 10 | rs141491107 | T | G | 0.1218 | 0.0255 | 0.075382 | 1.83E-06 | 39.21552 |
| 11 | rs2991233 | C | T | 0.1159 | 0.0225 | 0.926609 | 2.45E-07 | 44.44426 |
| 12 | rs34279440 | T | C | 0.286 | 0.0552 | 0.028888 | 2.23E-07 | 18.11587 |
| 13 | rs35872615 | G | T | 0.1923 | 0.0414 | 0.050787 | 3.36E-06 | 24.15449 |
| 14 | rs3846281 | C | A | 0.0588 | 0.0128 | 0.395766 | 4.36E-06 | 78.12467 |
| 15 | rs4398474 | T | C | 0.0699 | 0.0152 | 0.075815 | 4.11E-06 | 65.7892 |
| 16 | rs5011787 | A | T | 0.1171 | 0.0256 | 0.268095 | 4.89E-06 | 39.06234 |
| 17 | rs55958050 | C | G | 0.3625 | 0.0726 | 0.016131 | 5.93E-07 | 13.77405 |
| 18 | rs55958053 | G | T | -0.1064 | 0.0227 | 0.108777 | 2.72E-06 | 44.05268 |
| 19 | rs563792917 | C | G | -0.2991 | 0.0643 | 0.01044 | 3.28E-06 | 15.55203 |
| 20 | rs72774704 | T | G | -0.0669 | 0.0143 | 0.250898 | 2.92E-06 | 69.92978 |
| 21 | rs7582922 | C | T | 0.0507 | 0.011 | 0.569733 | 4.02E-06 | 90.90871 |
| 22 | rs76548953 | C | T | 0.3131 | 0.0674 | 0.019821 | 3.42E-06 | 14.83673 |
| 23 | rs77581913 | T | C | 0.3422 | 0.0739 | 0.015373 | 3.68E-06 | 13.53174 |
| 24 | rs77694071 | A | G | -0.0508 | 0.0109 | 0.37658 | 3.29E-06 | 91.74273 |

SNPs for DU

|  | SNP | Effect allele | Other allele | β | SE | EAF | P value | F-statistic |
| --- | --- | --- | --- | --- | --- | --- | --- | --- |
| 1 | rs1002891 | T | A | 0.125 | 0.0273 | 0.36435 | 4.51E-06 | 36.62986 |
| 2 | rs56283838 | A | G | 0.151 | 0.0303 | 0.262042 | 5.54E-07 | 33.00314 |
| 3 | rs79972697 | T | C | 0.194 | 0.0425 | 0.107557 | 4.99E-06 | 23.5293 |
| 4 | rs11723101 | C | A | 0.15 | 0.0308 | 0.237736 | 1.09E-06 | 32.46737 |
| 5 | rs3899063 | T | C | 0.176 | 0.0376 | 0.142472 | 2.95E-06 | 26.59561 |
| 6 | rs17053574 | C | G | 0.27 | 0.0587 | 0.054051 | 4.27E-06 | 17.03569 |
| 7 | rs117586568 | G | A | 0.386 | 0.0832 | 0.027328 | 3.54E-06 | 12.01917 |
| 8 | rs146446626 | G | T | 0.305 | 0.0653 | 0.047208 | 3.10E-06 | 15.31386 |

SNPs for IBS

|  | SNP | Effect allele | Other allele | β | SE | EAF | P value | F-statistic |
| --- | --- | --- | --- | --- | --- | --- | --- | --- |
| 1 | rs10156602 | G | A | -0.0417 | 0.0071 | 0.367 | 4.36E-09 | 140.8445 |
| 2 | rs10268606 | C | T | -0.037 | 0.0075 | 0.296 | 8.36E-07 | 133.3328 |
| 3 | rs10437560 | A | G | -0.0368 | 0.0078 | 0.6035 | 2.65E-06 | 128.2046 |
| 4 | rs10917847 | G | A | -0.0332 | 0.007 | 0.5344 | 1.87E-06 | 142.8566 |
| 5 | rs111491723 | C | A | -0.1153 | 0.0249 | 0.024 | 3.68E-06 | 40.16048 |
| 6 | rs112215352 | G | A | 0.0794 | 0.0171 | 0.0458 | 3.19E-06 | 58.47929 |
| 7 | rs11656412 | A | G | 0.0348 | 0.007 | 0.6096 | 7.78E-07 | 142.8566 |
| 8 | rs117654599 | A | T | 0.1693 | 0.0357 | 0.0128 | 2.07E-06 | 28.01109 |
| 9 | rs12358694 | G | A | 0.0328 | 0.0071 | 0.4663 | 4.47E-06 | 140.8445 |
| 10 | rs12464541 | C | T | 0.0421 | 0.0085 | 0.1999 | 7.27E-07 | 117.6466 |
| 11 | rs1248825 | C | A | -0.0444 | 0.0073 | 0.6747 | 1.20E-09 | 136.9857 |
| 12 | rs12530635 | C | G | 0.0338 | 0.0074 | 0.666 | 4.74E-06 | 135.1346 |
| 13 | rs12549729 | G | T | -0.0475 | 0.0086 | 0.6102 | 2.88E-08 | 116.2786 |
| 14 | rs12692627 | A | G | -0.0373 | 0.0076 | 0.2838 | 8.31E-07 | 131.5784 |
| 15 | rs12727764 | T | G | -0.0437 | 0.0093 | 0.1602 | 2.95E-06 | 107.5264 |
| 16 | rs1280622 | A | C | 0.0322 | 0.0069 | 0.5899 | 3.24E-06 | 144.9269 |
| 17 | rs12974306 | T | G | -0.0333 | 0.0069 | 0.483 | 1.35E-06 | 144.9269 |
| 18 | rs13031167 | T | G | 0.0826 | 0.0171 | 0.0896 | 1.31E-06 | 58.47929 |
| 19 | rs13251888 | A | G | 0.0747 | 0.0163 | 0.0551 | 4.83E-06 | 61.34944 |
| 20 | rs13321176 | G | T | -0.0478 | 0.0092 | 0.1702 | 1.80E-07 | 108.6952 |
| 21 | rs1631779 | A | C | -0.0611 | 0.0127 | 0.0788 | 1.40E-06 | 78.73983 |
| 22 | rs17210284 | C | T | 0.037 | 0.0081 | 0.231 | 4.78E-06 | 123.4563 |
| 23 | rs17331469 | T | G | 0.0473 | 0.0099 | 0.1368 | 2.01E-06 | 101.0097 |
| 24 | rs183066057 | G | A | 0.0945 | 0.0201 | 0.0469 | 2.48E-06 | 49.75104 |
| 25 | rs189713900 | G | A | 0.1528 | 0.0315 | 0.0156 | 1.18E-06 | 31.7459 |
| 26 | rs20551 | G | A | 0.0375 | 0.0075 | 0.2851 | 6.16E-07 | 133.3328 |
| 27 | rs2123209 | T | C | 0.0509 | 0.011 | 0.1094 | 3.33E-06 | 90.90872 |
| 28 | rs2197542 | T | G | 0.0484 | 0.0105 | 0.1232 | 3.64E-06 | 95.2377 |
| 29 | rs2728934 | G | A | 0.0374 | 0.0079 | 0.7379 | 2.09E-06 | 126.5818 |
| 30 | rs2731562 | A | C | -0.0317 | 0.0069 | 0.5117 | 3.66E-06 | 144.9269 |
| 31 | rs2828078 | A | G | -0.0467 | 0.0102 | 0.1428 | 4.45E-06 | 98.03881 |
| 32 | rs3096652 | A | G | -0.0326 | 0.007 | 0.5029 | 3.03E-06 | 142.8566 |
| 33 | rs35418299 | G | A | -0.0311 | 0.0068 | 0.4942 | 4.76E-06 | 147.0582 |
| 34 | rs4943187 | C | G | -0.0795 | 0.0174 | 0.9516 | 4.71E-06 | 57.47103 |
| 35 | rs5009448 | C | T | 0.0366 | 0.0079 | 0.7227 | 3.25E-06 | 126.5818 |
| 36 | rs5017929 | G | A | -0.0387 | 0.0075 | 0.2991 | 2.12E-07 | 133.3328 |
| 37 | rs56246023 | T | G | -0.0584 | 0.0123 | 0.1328 | 2.02E-06 | 81.30048 |
| 38 | rs56884006 | C | T | 0.0433 | 0.0092 | 0.2217 | 2.60E-06 | 108.6952 |
| 39 | rs57810551 | G | T | 0.0336 | 0.0071 | 0.3609 | 2.29E-06 | 140.8445 |
| 40 | rs6471719 | C | T | 0.0367 | 0.0076 | 0.2786 | 1.43E-06 | 131.5784 |
| 41 | rs6696068 | T | G | -0.035 | 0.007 | 0.3891 | 5.80E-07 | 142.8566 |
| 42 | rs6887323 | A | G | -0.0334 | 0.0069 | 0.5418 | 1.31E-06 | 144.9269 |
| 43 | rs72811605 | G | A | -0.0773 | 0.0166 | 0.0577 | 3.29E-06 | 60.24072 |
| 44 | rs7326447 | T | C | 0.106 | 0.0224 | 0.0261 | 2.30E-06 | 44.64267 |
| 45 | rs75963624 | A | G | 0.1854 | 0.0405 | 0.0099 | 4.77E-06 | 24.69126 |
| 46 | rs7751504 | T | C | -0.0328 | 0.007 | 0.3822 | 2.96E-06 | 142.8566 |
| 47 | rs7857016 | A | T | 0.0334 | 0.0069 | 0.4111 | 1.45E-06 | 144.9269 |
| 48 | rs8106322 | G | A | 0.0394 | 0.0075 | 0.354 | 1.45E-07 | 133.3328 |
| 49 | rs940468 | C | T | -0.0328 | 0.007 | 0.4248 | 2.77E-06 | 142.8566 |
| 50 | rs9513519 | G | A | -0.0388 | 0.007 | 0.3832 | 3.09E-08 | 142.8566 |
| 51 | rs996762 | G | C | -0.0499 | 0.0094 | 0.8424 | 1.12E-07 | 106.3825 |

SNPs for CD

|  | SNP | Effect allele | Other allele | β | SE | EAF | P value | F-statistic |
| --- | --- | --- | --- | --- | --- | --- | --- | --- |
| 1 | rs11209026 | A | G | 0.861114 | 0.099063 | 0.933019 | 3.92E-18 | 10.09452 |
| 2 | rs11255939 | C | T | 0.225183 | 0.046174 | 0.725246 | 9.67E-07 | 21.65722 |
| 3 | rs11948761 | T | A | -0.20089 | 0.041246 | 0.642972 | 1.09E-06 | 24.24468 |
| 4 | rs12264698 | A | G | -0.19844 | 0.042553 | 0.687213 | 2.82E-06 | 23.49976 |
| 5 | rs12555156 | A | G | 0.247519 | 0.054298 | 0.81769 | 4.16E-06 | 18.41684 |
| 6 | rs1887428 | C | G | 0.218928 | 0.040791 | 0.374702 | 5.97E-08 | 24.51538 |
| 7 | rs3792112 | T | C | -0.19771 | 0.039825 | 0.479269 | 6.40E-07 | 25.10974 |
| 8 | rs5743292 | A | T | -0.61226 | 0.086536 | 0.96346 | 1.85E-12 | 11.55583 |
| 9 | rs62234591 | C | T | -0.24606 | 0.050941 | 0.835901 | 1.17E-06 | 19.63044 |
| 10 | rs9611131 | C | T | 0.279505 | 0.060216 | 0.854768 | 3.01E-06 | 16.6068 |

SNPs for UC

|  | SNP | Effect allele | Other allele | β | SE | EAF | P value | F-statistic |
| --- | --- | --- | --- | --- | --- | --- | --- | --- |
| 1 | rs116394196 | C | T | -0.54211 | 0.117675 | 0.987292 | 3.46E-06 | 28.91596 |
| 2 | rs11692189 | A | G | -0.14299 | 0.031202 | 0.659801 | 4.63E-06 | 1567.435 |
| 3 | rs117675482 | G | A | 0.377685 | 0.079415 | 0.953353 | 1.80E-06 | 88.27474 |
| 4 | rs12064796 | A | G | 0.172799 | 0.030181 | 0.496418 | 8.75E-09 | 1109.66 |
| 5 | rs12209676 | G | A | -0.21458 | 0.043203 | 0.87534 | 8.63E-07 | 502.7064 |
| 6 | rs13115670 | A | G | -0.31594 | 0.067581 | 0.957145 | 2.60E-06 | 148.2395 |
| 7 | rs138036713 | A | C | -0.65538 | 0.141371 | 0.990867 | 3.32E-06 | 16.46811 |
| 8 | rs140388167 | G | T | -0.39648 | 0.087236 | 0.976222 | 4.34E-06 | 72.92339 |
| 9 | rs17657434 | G | A | -0.17909 | 0.039077 | 0.837682 | 4.63E-06 | 797.8297 |
| 10 | rs192541131 | A | C | 0.702065 | 0.152434 | 0.982878 | 3.23E-06 | 13.30946 |
| 11 | rs208346 | T | G | -0.14526 | 0.030399 | 0.606207 | 1.61E-06 | 1558.958 |
| 12 | rs2177011 | G | A | -0.15382 | 0.032005 | 0.695013 | 1.18E-06 | 1320.447 |
| 13 | rs2245950 | A | G | 0.1825 | 0.031718 | 0.640832 | 1.19E-08 | 946.6116 |
| 14 | rs35448556 | T | C | 0.221992 | 0.0484 | 0.878106 | 4.00E-06 | 419.2544 |
| 15 | rs4676408 | A | G | -0.14543 | 0.030192 | 0.478896 | 1.48E-06 | 1566.026 |
| 16 | rs55925810 | C | T | -0.15037 | 0.030146 | 0.475434 | 5.38E-07 | 1466.998 |
| 17 | rs6017342 | C | A | -0.17285 | 0.029995 | 0.48459 | 6.16E-09 | 1115.859 |
| 18 | rs62206995 | T | C | -0.38715 | 0.085516 | 0.974627 | 4.96E-06 | 78.016 |
| 19 | rs7035464 | G | A | -0.17997 | 0.035859 | 0.255746 | 4.37E-07 | 860.9981 |
| 20 | rs72704433 | C | T | -0.14529 | 0.031652 | 0.682071 | 3.79E-06 | 1496.734 |
| 21 | rs73370726 | T | C | 0.271215 | 0.054469 | 0.903146 | 5.91E-07 | 249.5869 |
| 22 | rs73647618 | A | G | 1.396137 | 0.294611 | 0.99385 | 1.84E-06 | 1.741375 |
| 23 | rs7553638 | G | A | 0.2099 | 0.02988 | 0.503708 | 1.68E-12 | 759.6235 |
| 24 | rs75698143 | A | G | 0.533182 | 0.110459 | 0.973764 | 1.22E-06 | 31.8452 |
| 25 | rs8099013 | C | T | 0.148545 | 0.030095 | 0.43385 | 3.84E-07 | 1505.855 |
| 26 | rs990107 | G | T | 0.162236 | 0.029958 | 0.429033 | 5.44E-08 | 1268.222 |

SNPs for NGE

|  | SNP | Effect allele | Other allele | β | SE | EAF | P value | F-statistic |
| --- | --- | --- | --- | --- | --- | --- | --- | --- |
| 1 | rs1015088 | A | G | -0.07043 | 0.01513 | 0.272474 | 3.24E-06 | 13324.6 |
| 2 | rs11159356 | G | A | 0.06849 | 0.013514 | 0.48433 | 4.02E-07 | 15774.4 |
| 3 | rs118154384 | T | C | 0.459404 | 0.096317 | 0.005297 | 1.85E-06 | 49.19311 |
| 4 | rs141980601 | T | C | -0.18353 | 0.039935 | 0.029517 | 4.31E-06 | 743.4276 |
| 5 | rs143680078 | G | A | 0.289457 | 0.062798 | 0.012037 | 4.04E-06 | 190.0574 |
| 6 | rs148228169 | C | G | 0.296938 | 0.065752 | 0.011119 | 6.30E-06 | 172.4888 |
| 7 | rs2188950 | A | G | 0.094647 | 0.020254 | 0.127088 | 2.97E-06 | 5511.54 |
| 8 | rs2333345 | G | A | 0.068269 | 0.014085 | 0.361552 | 1.25E-06 | 15233.2 |
| 9 | rs2941949 | C | G | 0.099361 | 0.020508 | 0.122587 | 1.27E-06 | 4939.156 |
| 10 | rs4589405 | A | G | -0.14602 | 0.028751 | 0.060238 | 3.80E-07 | 1631.249 |
| 11 | rs4911532 | T | C | -0.06222 | 0.013615 | 0.426495 | 4.87E-06 | 18969.67 |
| 12 | rs62036857 | T | G | 0.189737 | 0.038985 | 0.031145 | 1.13E-06 | 712.5264 |
| 13 | rs62244135 | C | T | 0.160486 | 0.034483 | 0.040534 | 3.26E-06 | 1125.936 |

SNPs for CP

|  | SNP | Effect allele | Other allele | β | SE | EAF | P value | F-statistic |
| --- | --- | --- | --- | --- | --- | --- | --- | --- |
| 1 | rs10139484 | T | G | -0.0526 | 0.0114 | 0.545353 | 4.35E-06 | 87.71887 |
| 2 | rs10223052 | G | A | 0.0546 | 0.0119 | 0.566463 | 4.84E-06 | 84.03321 |
| 3 | rs11648475 | G | A | 0.0546 | 0.0114 | 0.48264 | 1.75E-06 | 87.71887 |
| 4 | rs12934865 | T | C | 0.0558 | 0.0115 | 0.460637 | 1.20E-06 | 86.9561 |
| 5 | rs1376767 | C | A | -0.0706 | 0.014 | 0.756497 | 4.72E-07 | 71.42822 |
| 6 | rs186529831 | T | C | -0.2466 | 0.0512 | 0.01975 | 1.50E-06 | 19.53116 |
| 7 | rs2354178 | A | G | -0.0592 | 0.0128 | 0.75465 | 4.01E-06 | 78.12462 |
| 8 | rs4083752 | A | G | 0.0565 | 0.0118 | 0.455926 | 1.70E-06 | 84.74535 |
| 9 | rs55694472 | T | C | 0.1641 | 0.0286 | 0.043318 | 9.91E-09 | 34.96487 |
| 10 | rs56029819 | C | T | 0.0757 | 0.0162 | 0.150442 | 2.83E-06 | 61.7281 |
| 11 | rs5757641 | G | A | -0.0585 | 0.0121 | 0.363219 | 1.28E-06 | 82.64423 |
| 12 | rs59674167 | T | C | -0.0718 | 0.0148 | 0.19228 | 1.17E-06 | 67.56724 |
| 13 | rs616812 | C | T | -0.0631 | 0.0135 | 0.725106 | 3.16E-06 | 74.07371 |
| 14 | rs74287578 | C | A | -0.1576 | 0.0323 | 0.096772 | 1.05E-06 | 30.9596 |
| 15 | rs75271724 | G | T | 0.2218 | 0.0456 | 0.016711 | 1.14E-06 | 21.92972 |
| 16 | rs79086497 | A | G | -0.0811 | 0.0176 | 0.120788 | 3.93E-06 | 56.81791 |

SS = Sjögren's syndrome, GU = Gastric ulcer, DU = Duodenal ulcer, IBS = Irritable bowel syndrome, CD = Crohn’s disease, UC = Ulcerative colitis, NGE = Non‑infectious gastroenteritis, CP = Constipation, SNP = Single Nucleotide Polymorphism, β = Beta Coefficient, SE = Standard Error, EAF = Effect Allele Frequency, SNP = Single Nucleotide Polymorphism, β = Beta Coefficient, SE =Standard Error, EAF = Effect Allele Frequency.
