# Supplementary material for: Bidirectional causal associations between Sjögren syndrome and gastrointestinal disorders: A two-sample Mendelian randomization study
Source: Medicine (Baltimore). 2026 Sep 25;105(39):e50935. doi: 10.1097/MD.0000000000050935 (PMC13619203; doi:10.1097/MD.0000000000050935)
Supplement: Supplementary file 2 [file medi-105-e50935-s002.docx]

**Supplementary Table 2** Sensitivity analysis of SS on seven gastrointestinal diseases.

| Exposures | Outcomes | Heterogeneity test | | | | Pleiotropy test | | | |
| --- | --- | --- | --- | --- | --- | --- | --- | --- | --- |
|  |  | IVW | | MR-Egger | | MR-Egger intercept | | MR PRESSO | |
|  |  | Q | P value | Q | P value | intercept | pval | RSSobs | P value |
| SS | GU | 14.239 | 0.357 | 12.209 | 0.429 | -0.010 | 0.183 | 16.702 | 0.361 |
| SS | DU | 12.583 | 0.400 | 12.539 | 0.324 | 0.003 | 0.849 | 13.473 | 0.540 |
| SS | IBS | 18.499 | 0.139 | 14.939 | 0.245 | 0.007 | 0.117 | 57.258 | 0.293 |
| SS | CD | 21.717 | 0.060 | 16.276 | 0.179 | -0.053 | 0.068 | 42.576 | 0.079 |
| SS | UC | 16.672 | 0.162 | 13.329 | 0.272 | 0.017 | 0.440 | 23.842 | 0.243 |
| SS | NGE | 18.672 | 0.097 | 16.691 | 0.117 | -0.031 | 0.125 | 27.212 | 0.177 |
| SS | CP | 16.542 | 0.221 | 16.407 | 0.173 | 0.002 | 0.759 | 21.029 | 0.251 |

SS = Sjögren's syndrome; GU = gastric ulcer; DU = duodenal ulcer; IBS = irritable bowel syndrome, CD = Crohn’s disease; UC = ulcerative colitis; NGE = non-infectious gastroenteritis; CP = constipation; MR = mendelian randomization; IVW = inverse variance weighted; Q = Cochran’s Q statistic; PRESSO = Pleiotropy RESidual Sum and Outlier; RSSobs = Observed residual sum of squares.
