# Supplementary material for: Bidirectional causal associations between Sjögren syndrome and gastrointestinal disorders: A two-sample Mendelian randomization study
Source: Medicine (Baltimore). 2026 Sep 25;105(39):e50935. doi: 10.1097/MD.0000000000050935 (PMC13619203; doi:10.1097/MD.0000000000050935)
Supplement: Supplementary file 3 [file medi-105-e50935-s003.docx]

**Supplementary Table 3** Sensitivity analysis of seven gastrointestinal diseases on SS.

| Exposures | Outcomes | Heterogeneity test | | | | Pleiotropy test | | | |
| --- | --- | --- | --- | --- | --- | --- | --- | --- | --- |
|  |  | IVW | | MR-Egger | | MR-Egger intercept | | MR PRESSO | |
|  |  | Q | P value | Q | P value | intercept | pval | RSSobs | P value |
| GU | SS | 20.525 | 0.550 | 21.453 | 0.493 | -0.007 | 0.660 | 23.492 | 0.570 |
| DU | SS | 9.548 | 0.216 | 7.083 | 0.313 | -0.062 | 0.199 | 12.736 | 0.217 |
| IBS | SS | 54.029 | 0.255 | 53.185 | 0.316 | 0.018 | 0.159 | 57.258 | 0.293 |
| CD | SS | 4.802 | 0.851 | 4.785 | 0.780 | 0.003 | 0.898 | 5.684 | 0.862 |
| UC | SS | 37.292 | 0.054 | 32.728 | 0.110 | 0.023 | 0.080 | 43.603 | 0.056 |
| NGE | SS | 12.109 | 0.437 | 11.899 | 0.371 | 0.009 | 0.668 | 13.846 | 0.504 |
| CP | SS | 9.892 | 0.827 | 9.795 | 0.777 | 16.542 | 0.221 | 11.275 | 0.833 |

Abbreviations as defined in Supplementary Table 2.
